# Accuracy of imputation to whole-genome sequence in sheep

**DOI:** 10.1186/s12711-018-0443-5

**Published:** 2019-01-17

**Authors:** Sunduimijid Bolormaa, Amanda J. Chamberlain, Majid Khansefid, Paul Stothard, Andrew A. Swan, Brett Mason, Claire P. Prowse-Wilkins, Naomi Duijvesteijn, Nasir Moghaddar, Julius H. van der Werf, Hans D. Daetwyler, Iona M. MacLeod

**Affiliations:** 1 Agriculture Victoria, AgriBio, Centre for AgriBioscience, 5 Ring Rd, Bundoora, VIC 3083 Australia; 2 Cooperative Research Centre for Sheep Industry Innovation, Armidale, NSW 2351 Australia; 3 Faculty of Agricultural, Life and Environmental Sciences, University of Alberta, Edmonton, AB T6G 2R3 Canada; 4 Animal Genetics and Breeding Unit, University of New England, Armidale, NSW 2351 Australia; 5 School of Environmental and Rural Science, University of New England, Armidale, NSW 2351 Australia; 6 School of Applied Systems Biology, La Trobe University, Bundoora, VIC 3086 Australia

## Abstract

**Background:**

The use of whole-genome sequence (WGS) data for genomic prediction and association studies is highly desirable because the causal mutations should be present in the data. The sequencing of 935 sheep from a range of breeds provides the opportunity to impute sheep genotyped with single nucleotide polymorphism (SNP) arrays to WGS. This study evaluated the accuracy of imputation from SNP genotypes to WGS using this reference population of 935 sequenced sheep.

**Results:**

The accuracy of imputation from the Ovine Infinium^®^ HD BeadChip SNP (~ 500 k) to WGS was assessed for three target breeds: Merino, Poll Dorset and F1 Border Leicester × Merino. Imputation accuracy was highest for the Poll Dorset breed, although there were more Merino individuals in the sequenced reference population than Poll Dorset individuals. In addition, empirical imputation accuracies were higher (by up to 1.7%) when using larger multi-breed reference populations compared to using a smaller single-breed reference population. The mean accuracy of imputation across target breeds using the Minimac3 or the FImpute software was 0.94. The empirical imputation accuracy varied considerably across the genome; six chromosomes carried regions of one or more Mb with a mean imputation accuracy of < 0.7. Imputation accuracy in five variant annotation classes ranged from 0.87 (missense) up to 0.94 (intronic variants), where lower accuracy corresponded to higher proportions of rare alleles. The imputation quality statistic reported from Minimac3 (*R*^2^) had a clear positive relationship with the empirical imputation accuracy. Therefore, by first discarding imputed variants with an *R*^*2*^ below 0.4, the mean empirical accuracy across target breeds increased to 0.97. Although accuracy of genomic prediction was less affected by filtering on *R*^2^ in a multi-breed population of sheep with imputed WGS, the genomic heritability clearly tended to be lower when using variants with an *R*^2^ ≤ 0.4.

**Conclusions:**

The mean imputation accuracy was high for all target breeds and was increased by combining smaller breed sets into a multi-breed reference. We found that the Minimac3 software imputation quality statistic (*R*^2^) was a useful indicator of empirical imputation accuracy, enabling removal of very poorly imputed variants before downstream analyses.

**Electronic supplementary material:**

The online version of this article (10.1186/s12711-018-0443-5) contains supplementary material, which is available to authorized users.

## Background

The Australian sheep industry has embraced the application of genomics to enhance genetic improvement programs [1]. Already, more than 47,000 Australian sheep have been genotyped by using mainly low- (12 k) and medium-density (50 k) single nucleotide polymorphism (SNP) arrays [1]. Currently, the sheep industry uses real or imputed 50 k SNP genotypes for genomic prediction of breeding values. Imputation is the process of predicting unknown genotypes for animals genotyped at lower SNP density by using a reference set of animals genotyped at the higher SNP density.

Genomic prediction relies on strong linkage disequilibrium (LD) between causal variants for a given trait and the SNPs. Such LD might not exist when using the 50 k SNP array for prediction of more distantly related animals or prediction across breeds. Furthermore, SNPs on the 50 k SNP array were preselected to be (highly) polymorphic in the target breeds. This can result in rarer causal variants not being in strong LD with the standard 50 k SNPs, and thus their effects may not be captured in the genomic predictions. In contrast to SNP arrays, whole-genome sequence (WGS) data include all or at least many causal variants. Thus, genomic prediction with sequence data would not need to rely on LD with common SNPs on arrays. Although it is not yet economically feasible to sequence many thousands of animals, it is possible to impute sequence variants for animals that are already genotyped on standard SNP arrays if a reference population of sequenced animals is available [2]. There is now evidence in both cattle and sheep that the accuracy of genomic prediction can be increased by combining more predictive variants from imputed sequence data with a standard SNP array, particularly for across-breed prediction or prediction of animals with low relationships to the reference animals [3–8]. In order to develop the opportunity to exploit imputed WGS data in sheep, a joint collaboration between SheepGenomesDB (www.sheepgenomesdb.org) and the Sheep CRC project has sequenced a reference population of almost 1000 sheep [9]. A range of worldwide breeds were sequenced, with the largest proportion being Merino and Merino crosses.

To date in sheep, there has been no evaluation of the accuracy of imputation to whole-genome sequence data (WGS). Thus, the main objective of our study was to evaluate a practical approach to achieve highly accurate imputed WGS in a range of purebred and crossbred sheep, that would easily scale up to imputing more than 60,000 individuals. A range of software programs is available to carry out imputation (e.g. Beagle [10], FastPhase [11], Impute2 [12], FImpute [13], and Minimac3 [14]), each one implementing a different algorithm that can affect both computational speed and imputation accuracy. Beagle and FImpute have been widely used in livestock populations and consistently achieve high imputation accuracies [13, 15]. Recently, Minimac3 was shown to be highly accurate and computationally efficient for imputation to sequence in human populations [14] and also compared favourably with FImpute in cattle [16]. Therefore, we evaluated the accuracy of imputed WGS using the FImpute and Minimac3 software when the starting point from which to impute to WGS was high-density (“HD”: ~ 500,000) SNP genotypes. We also evaluated the accuracy of imputation to WGS when the starting point was low-density SNP genotypes (OvineLD: 12 k SNPs). In the sequenced reference population, many breeds are represented, with some having few individuals and others having many individuals. Thus, we validated the accuracy of imputed WGS in three target (two purebred and one crossbred) populations using either all or a subset of the sequenced reference set. In addition, we investigated local imputation accuracy along each chromosome to identify regions in the ovine genome that are poorly imputed, possibly due to large segmental repeat regions and/or errors in the reference genome. Furthermore, we demonstrated the impact of using different Minimac3 quality (*R*^2^) thresholds, as a proxy for detecting poorly or well-imputed variants, on the accuracy of genomic prediction for three traits in a large multi-breed population.

## Methods

### Reference genotype data

A reference set of WGS data was available for 935 animals representing multiple breeds and crosses (see Additional file 1: Table S1) from across the world, with approximately 10 × average read depth [9]. The raw fastq WGS dataset was processed using an in-house pipeline to undertake the following quality control with the QUADTrim program (https://bitbucket.org/arobinson/quadtrim): adapter sequences and bases for which the qscore was < 20 were trimmed from the 3’ and 5’ end of reads; reads with more than three missed base calls and shorter than 50 reads were removed; bases for which the qscore was < 20 for three consecutive bases were trimmed from reads; and entire reads with a mean score < 20 were removed. Then, the reads were aligned to the OAR (*Ovis aries*) 3.1 ovine reference genome using the Burrows-Wheeler Aligner [17]. Duplicates were removed using Samtools [18] and a local realignment around indels was performed using the GATK software [19]. Variant calling for SNPs and short insertions and deletions (indels) was carried out simultaneously for all 935 sequenced animals using a multi-sample variant calling pipeline that was implemented with the *mpileup* module of SAMtools [18], as described by Daetwyler et al. [9], which yielded polymorphisms at 52,676,272 biallelic sites. All variants were annotated following the pipeline developed by Grant et al. [20].

We evaluated imputation accuracy using three reference sets: MER = 117 pure Merino animals, EUR = 726 sheep representing only European breeds, and ALL = 935 animals representing breeds from Europe, Africa, Asia, America and the Middle East. For all three reference sets, prior to imputation, we removed variants with minor allele counts < 5 (across all reference animals) and variants with more than two alleles. This resulted in 42,249,885, 39,844,235, and 30,660,937 polymorphic variants in the ALL, EUR, and MER reference sets, respectively.

### Target genotype data

The accuracy of the imputed WGS was assessed in sequenced animals by first masking all their sequence genotypes, except for those that overlapped with variants in either the HD or OvineLD SNP arrays. To calculate imputation accuracy, we used the following three target populations with fivefold cross-validation: MER = 117 pure Australian Merinos, PD = 29 pure Poll Dorsets, and F1 = 59 Merino × Border Leicester crossbreds. For each of the fivefold validation groups, the WGS genotypes for 20% of the MER, PD, or F1 animals were reduced to either HD or OvineLD SNPs, and then these animals were used as a target set for imputation to the sequence level. This ensured that a large proportion of animals of the same breeds (80% of the total) were still available in the reference population. The positions of the Ovine Infinium^®^ HD BeadChip SNPs (developed under the auspices of the International Sheep Genomics Consortium) that mapped to the OAR 3.1 build of the ovine reference genome [21] were used to prepare these target HD genotype sets. Likewise, we used the OAR 3.1 positions for the OvineLD BeadChip [22]. For the imputation test from OvineLD SNP to WGS, we used the three target sets above but only with the ALL reference set.

### Imputation to whole-genome sequence

The imputation test using ALL reference and MER target sets was done for the 26 ovine autosomes. For the other scenarios, to reduce computational requirements, we tested imputation accuracy on a representative set of six autosomal chromosomes (OAR for *Ovis aries*): 1, 5, 10, 15, 20 and 25.

#### Imputation from HD to WGS

After masking all the sequence genotypes in the target fivefold cross-validation set, except for those that overlapped with the HD SNPs, imputation was carried out directly from HD genotypes to WGS variants using two software methods: FImpute (version 2.2; [13]) and Minimac3 (version 2.0.1; [23]). Minimac3 requires pre-phased genotypes in both the reference (WGS) and target sets, for which the Eagle software (version 2.3; [24]) was used. Default parameters were used for Eagle and Minimac3. The Minimac3 software provides the most likely genotypes (coded as 0, 1 and 2 for homozygous, heterozygous and alternative homozygous animals, respectively), as well as the predicted allele dosage (continuously distributed values ranging from 0 to 2) for imputed variants. Neither Eagle nor Minimac3 consider pedigree information to infer haplotypes or missing genotypes. FImpute can use pedigree and population-based information to infer haplotypes and missing genotypes. However, in general, sheep pedigrees are shallow and error prone and, thus, we implemented FImpute without pedigree information and applied default parameters otherwise. Then, the imputed WGS genotypes were compared to their real genotypes, except for the variants that overlapped with the HD SNPs.

#### Imputation from OvineLD to WGS

After masking all the sequence genotypes in the target fivefold cross-validation set, except for those that overlapped with the OvineLD SNPs, imputation from OvineLD SNPs to WGS was performed in three sequential stages:

1. The OvineLD SNPs (~ 12,223) were imputed to the OvineSNP50 Beadchip (Illumina Inc., San Diego, CA, USA) SNPs, using FImpute and a reference population of 1933 animals that were genotyped directly with 38,378 SNPs. The animals from this reference population were from multiple breeds, including 555 pure Merino, 36 Border Leicester, 19 pure Suffolk, 18 pure Poll Dorset, 11 pure Texel, and other minor pure breeds and crossbreeds [e.g. F1 crosses between PD × MER (86) and BL × MER (9)].
2. The 50 k imputed genotypes were then imputed to HD SNPs using Eagle pre-phasing and Minimac3 (as described for imputation from HD to WGS), using the same reference population of 1933 animals, which were also genotyped directly with the HD SNPs.
3. The imputed HD genotypes were imputed to WGS variants using Eagle pre-phasing and Minimac3.

Imputed WGS genotypes were compared to their real genotypes after removing the overlapping variants in the imputed HD genotypes, as for imputation from HD to WGS.

### Assessing imputation accuracy

The empirical accuracy of imputation was assessed as the correlation between real and imputed genotypes for all sequence variants (excluding HD SNPs) across the five-fold cross-validations. The mean imputation accuracy was also calculated per chromosome and in non-overlapping 1-Mb windows across each chromosome to identify genomic regions that were difficult to impute well because of either large duplicated segments and/or poor reference map quality. These genomic regional tests were carried out across all autosomes, using Minimac3 for the MER target with the ALL reference scenario.

Empirical imputation accuracies were also compared with the Minimac3 *R*^2^ quality statistic that is an estimate of imputation accuracy based on the concept that poorly imputed genotype counts will shrink towards their expectations across all individuals, based on population allele frequencies (https://genome.sph.umich.edu/wiki/Minimac3_Info_File). This comparison was made by evaluating the mean empirical imputation accuracies for groups of variants that were allocated to 100 bins based on their *R*^2^ value: 0 to 0.01, 0.01 to 0.02, etc.

We tested the effect of imposing a minimum Minimac3 *R*^2^ threshold on empirical imputation accuracy. That is, prior to calculating the correlation between imputed and sequenced variants, variants were discarded if their *R*^2^ was lower than a given threshold (i.e. variants were retained with *R*^2^ > 0.4 = “thr4” or *R*^2^ > 0.8 = “thr8”).

### Annotated variants

Variants in the ALL set of sheep reference genomes were annotated following [20]. The Ensembl (version 87; http://dec2016.archive.ensembl.org/index.html) functional annotation categories included five key annotation groupings: intergenic, intronic, 5 kb up- or down-stream of genes, missense, and UTR 3’ and 5’ ends. The mean empirical imputation accuracy was calculated for each of these five classes using the MER target set with the ALL reference set. We also assessed the mean Minimac3 *R*^2^ and MAF distributions for variants in each class.

### Impact of imputation accuracy (Minimac3 *R*^2^) on genomic prediction

We evaluated the impact of using poorly or well imputed genotypes on the accuracy of genomic prediction, using Minimac3 *R*^2^ as a proxy for empirical imputation accuracy. This was evaluated for three traits: carcass fat depth at C site (ccfat), post-weaning eye muscle depth (pemd), and post-weaning weight (pwt).

#### Phenotypes

The data for this genomic prediction was previously described by Khansefid et al. [3]. Briefly, the reference and validation sets included up to 20,403 animals across these three traits. The reference sets included 1910 pure Merinos (MER), 1360 Poll Dorset (PD), 355 pure Border Leicester (BL), 1360 PD × MER crosses, 619 BL × MER crosses, 703 pure White Suffolk and its crossbred animals, and other minor breeds, their crosses and composite animals. Animals in the validation sets were purebred Merinos. None of the validation animals shared paternal half-sibs in the reference population. Phenotypes were obtained from Australia’s Sheep Genetics industry genetic evaluation database [1], and were processed by the AGBU sheep evaluation team (http://agbu.une.edu.au/sheep.html) including: pre-adjustment for various fixed effects (birth-rearing type, sex, and contemporary groups) and removal of animals with a phenotype more than 4 standard deviations from the mean. Phenotypes were also pre-corrected for the random genetic group effect and data source (as described in [3]). Trait definitions, number of records used in the reference and validation sets for each trait, raw means and standard deviations (prior to pre-adjustment) based on the genotyped and phenotyped animals are in Table 1.

**Table 1:** Numbers of phenotypes used in the reference and target sets for genomic prediction for each trait, means and standard deviations across all animals, and estimates of genomic heritability

Trait name (abbreviation, unit)	Number of phenotypes		Mean	SD	Heritability
	Reference set	Target set			
Fat depth C (ccfat, mm)	7635	912	4.0	2.3	0.18
Post-weaning eye muscle depth (pemd, mm)	9715	1766	25.4	4.9	0.22
Live weight measured post weaning (pwt, kg)	11,067	3118	42.2	7.6	0.21

#### Genotypes

We imputed the WGS genotypes using the EUR reference and Minimac3 with Eagle pre-phasing (following the description above) for ~ 47,000 Australian sheep, including all reference and validation animals described above. All animals had previously been genotyped with either the LD, 50 k or HD SNP arrays. Genotypes on the X chromosome were excluded.

For the 47,000 sheep, we did not have a direct estimate of the true accuracy of their imputed WGS. Therefore, we evaluated the impact on the accuracy on genomic prediction by using the Minimac3 *R*^2^ statistic as a proxy filter for poorly imputed variants. For this test, we used genotype sets of 50,000 variants (MAF > 0.01) that were randomly selected across the genome from the available WGS variants. We used three filters to select variant sets based on the Minimac3 *R*^2^: imputed variants with *R*^2^ ≤ 0.4 (thr0-4), *R*^2^ > 0.4 (thr4), and *R*^2^ > 0.8 (thr8), respectively.

For each of these three filters, five random sets of 50,000 sequence variants were selected while ensuring that each random set shared a similar allele frequency distribution (as an example, the allele frequency distribution for ccfat for each filter with five random sets is shown in Additional file 2: Figure S1). The mean distance between adjacent variants across each of the three filters matched expectations based on the total length of the imputed autosomal chromosomes (mean distance between adjacent variants was 48.99 kb for thr0-4, 48.87 kb for thr4, and 48.78 kb for thr8).

Genomic prediction analyses based on genotypes for each of the 50,000 variant sets were performed using the BayesR method [25], which jointly fits the effects of all SNPs and assumes that SNP effects are from a mixture of four normal distributions with variances equal to 0, 0.01, 0.1 or 1% of the genetic variance, respectively [25]. Gibbs sampling was used for sampling from the posterior distributions of the parameters, running 40,000 iterations with 20,000 iterations of burn-in. Five parallel chains were run for each trait and each of the five-random genotype sets per *R*^2^ filter (that is: 5 chains × 5 genotype sets = 25 analyses per trait for each filter). The SNP effects from each of the 25 BayesR analyses per trait and per *R*^2^ filter were then used to calculate genomic estimated breeding values (GEBV) for the Merino target set. The accuracy of genomic prediction was calculated as the correlation of the GEBV with the adjusted phenotype, divided by the square root of the heritability of the trait (${\text{h}}^{2}$). The latter was the mean of the estimated genomic heritability (shown in Table 1), which was estimated as the proportion of phenotypic variance that was explained by the 50,000 sequence variants. Estimates of genomic heritability and of the accuracy of GEBV were averaged across the five parallel Gibbs sampling chains run for each variant set per trait, giving five estimates for each of the five variant sets for each filter. The standard error of the accuracy of GEBV was estimated from the five randomly sampled independent genotype sets for each trait, i.e. as the standard deviation of the five accuracies divided by the square root of 5.

## Results

**Fig. 1:**
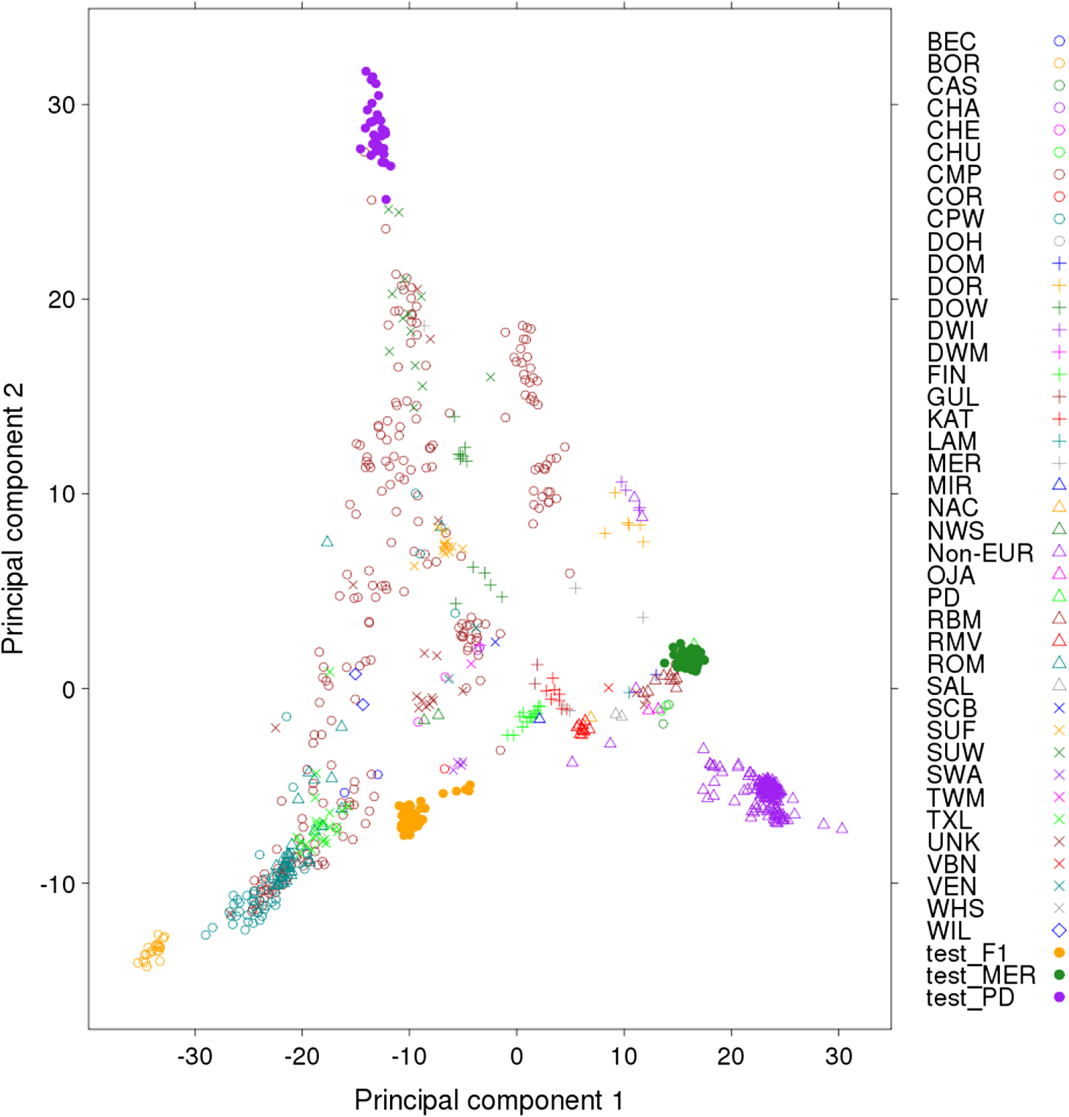
Principal component decompositions of the genomic relationship matrix constructed from whole-genome sequence genotypes pruned down to HD SNP genotypes for 935 sequenced animals. Description of breed names is in Additional file 1: Table S1. Filled circles represent animals tested in the imputation from the Merino (test_MER, green), Poll Dorset (test_PD, purple), and Merino × Border Leicester (test_F1, orange) breeds

All imputation accuracy results presented are based on imputation from the Ovine Infinium^®^ HD BeadChip SNP (~ 500 k) to WGS, except when stated otherwise. A principal component analysis (PCA) was used to show the genetic diversity of the sequenced animals that were allocated to reference and target validation sets (Fig. 1). The PCA was based on a genomic relationship matrix that was generated from their WGS variants pruned down to the HD SNP genotype set. Animals from the MER, PD and F1 breeds that were used as imputation target populations, appear as tight groups at opposite extremes of the first and second principal component axes.

### Minimac3 versus FImpute

**Fig. 2:**
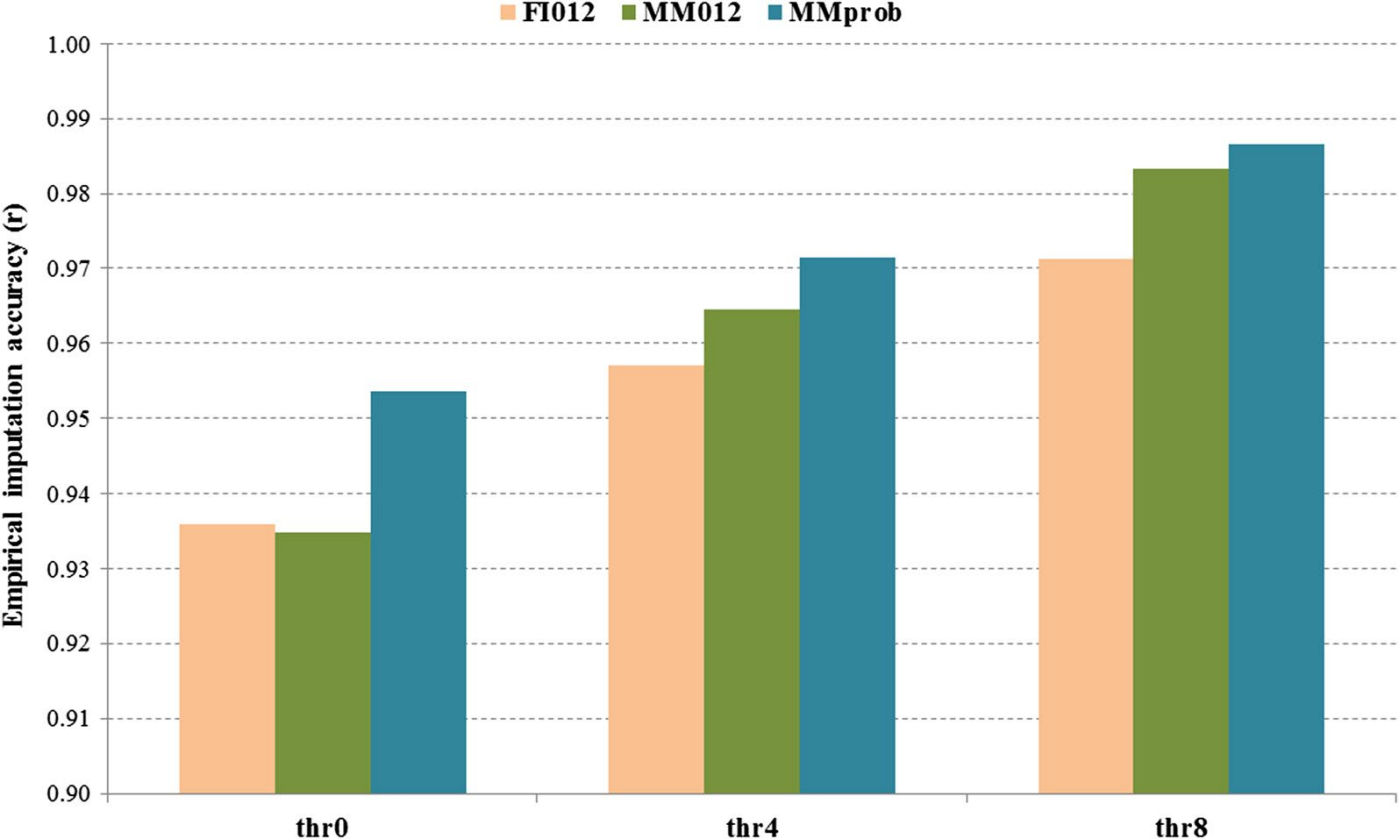
Empirical imputation accuracy of most likely whole-genome sequence genotypes based on FImpute (FI012) and Minimac3 (MM012) and of the Minimac3 allele dosage probability (MMprob) in a Merino (MER) target population, using a mixed breed reference set (‘ALL’). Imputation accuracy is before and after imposing Minimac3 *R*^2^ thresholds to filter the imputed sequence data: thr0 = no threshold, thr4 =* R*^2^ > 0.4, and thr8 = *R*^2^ > 0.8. Imputation accuracy was measured as the correlation between imputed and observed sequence genotypes in the target population

Figure 2 compares the empirical imputation accuracy obtained with the FImpute and Minimac3 imputation softwares when the ALL reference set was used to impute the MER target test set. For the comparison between the imputation methods, only the overlapping set of imputed variants from FImpute and Minimac3 imputation was compared because FImpute was not able to impute the region between 70 and 74 Mb on OAR10. Figure 2 also shows the increase in empirical imputation accuracy when the imputation quality statistic of Minimac3, *R*^2^, was used to retain variants more likely to be imputed well by applying three *R*^2^ thresholds: no threshold (thr0), *R*^2^ > 0.4 (thr4) and *R*^2^ > 0.8 (thr8). Imputation accuracies for Minimac3 and FImpute were assessed based on the most likely genotypes (‘MM012’ and ‘FI012’, coded as 0, 1 and 2 for homozygous, heterozygous and alternative homozygous genotypes, respectively) and also based on genotype probabilities obtained with Minimac3 (‘MMprob’: continuously distributed allele dosage values ranging from 0 to 2). The highest imputation accuracy was consistently achieved with MMprob, both before and after imposing an *R*^2^ filter but the difference between MMprob and MM012 was lower for *R*^2^ thr4 and thr8 filters (Fig. 2). For the most likely genotypes, MM012 and FI012, there was little difference between Minimac3 and FImpute without the *R*^2^ filter (thr0) but was slightly and consistently higher for MM012 than for FI012 after imposing a filter using *R*^2^ (Fig. 2, results shown only for using ALL as a reference and MER as a target set). The use of *R*^2^ computed using Minimac3 as a filter may, however, have introduced some bias against FImpute because some of the removed variants with low *R*^2^ may have been accurately imputed by FImpute. There was, however, no equivalent statistic available from FImpute.

**Fig. 3:**
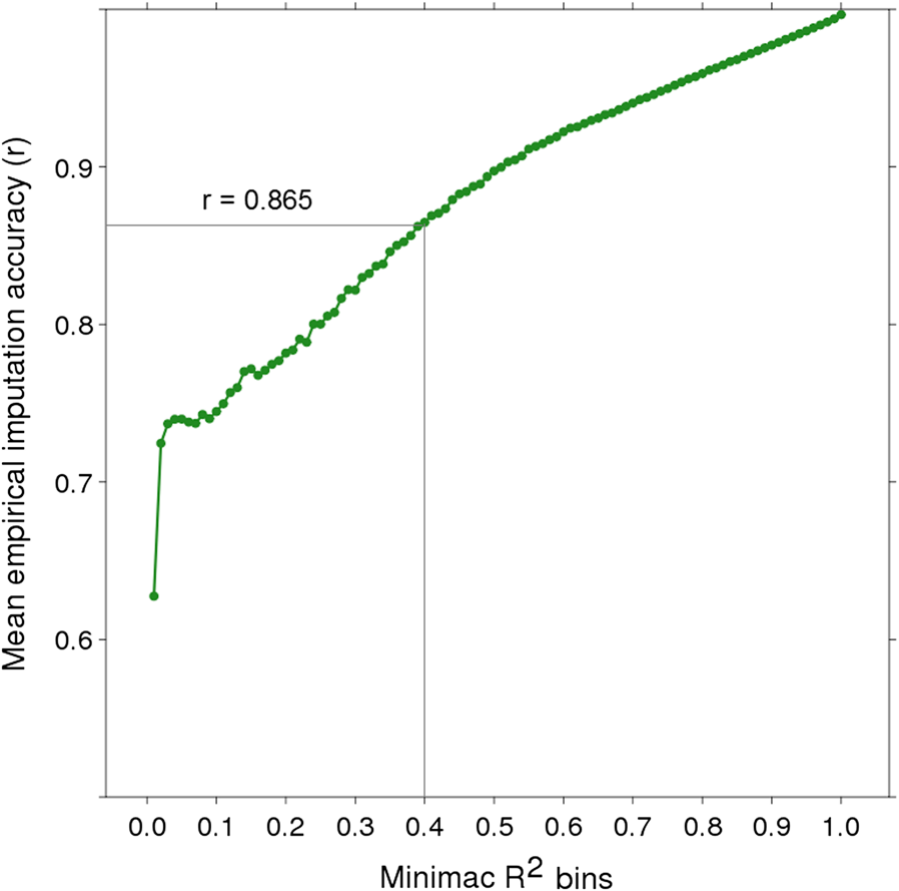
Mean empirical imputation accuracy of whole-genome sequence genotypes (Minimac3 most likely genotypes) by Minimac3 *R*^2^ of the imputed variants. The plot shows the relationship between variants grouped in *R*^*2*^ bins (x axis) and their mean empirical imputation accuracy for each bin (y axis). This relationship was evaluated in the Merino target (MER) set using a mixed breed reference set (ALL)

Our results also demonstrated a clear relationship between empirical imputation accuracy *R*^2^ when comparing the average empirical imputation accuracy for groups of variants allocated to *R*^2^ bins (Fig. 3). For example, when using the ALL reference and the MER target sets, the mean empirical imputation accuracy was 0.86 for variants with *R*^2^ between 0.40 and 0.41 compared to 0. 90 for variants with *R*^2^ between 0.50 and 0.51. This demonstrates that the Minimac3 *R*^2^ is a useful indicator of empirical imputation. This relationship was slightly stronger when a within-breed rather than the ALL breed reference was considered (e.g. the empirical imputation accuracy was 0.88 for variants with an *R*^2^ between 0.40 and 0.41 when using MER as reference and target).

We also estimated the mean imputation accuracy for groups of variants within a range of MAF and, generally, accuracy was lower for variants with low MAF (Table 2). The accuracy was a little higher for MMprob than for MM012 and FI012 across all levels of MAF.

**Table 2:** Imputation accuracy of whole-genome sequence genotypes in three minor allele frequency (MAF) bands in a Merino target population, using a mixed breed reference set (‘ALL’)

MAF band	FI^a^	MM012^b^	MMprob^c^
0.01–0.02^d^	0.856	0.858	0.886
0.02–0.03	0.871	0.877	0.902
0.03–0.04	0.883	0.889	0.913
0.05–0.06	0.892	0.898	0.920
0.06–0.07	0.902	0.906	0.927
0.06–0.08	0.913	0.916	0.936
0.08–0.10	0.922	0.923	0.942
0.10–0.50	0.927	0.922	0.945

^a^FImpute (FI012); ^b^Minimac3 (MM012) most likely genotypes; and ^c^Minimac3 allele dosage probability (MMprob); and ^d^lower MAF could not be evaluated because the number of animals in the target set was 117

Because differences in imputation accuracies between MM012, MMprob, and FI012 were small, hereafter, only results for the Minimac3 imputed most likely genotypes (MM012) will be presented.

### Imputation accuracy using different reference and target sets

In the MER target set, the accuracy of imputation using mixed breed reference sets (ALL or EUR) was slightly higher (for thr0) or the same (for thr4) than for the smaller MER single-breed reference (Table 3). Notably, using either the ALL or EUR reference set resulted in more than 2.3 million (M) extra variants imputed compared to using the MER reference set. The proportion of these extra variants that were polymorphic in the real MER sequence was 96%. However, these variants were found to have < 5 allele counts in the MER target population and therefore were discarded from the MER only imputation (see Methods section). We were able to recover some of the variants that were very rare in the single breed reference (MER) set by including a range of breeds and crosses for which the allele frequency was higher. Even after imposing the *R*^2^ thr4 filter, the ALL or EUR reference set had more than 0.8 M variants than MER reference set.

**Table 3:** Minimac3 imputation accuracy (*R*^2^) for variants before (thr0) or after (thr4) filtering based on *R*^*2*^ threshold (numbers of variants imputed in millions in brackets) in Merino target set animals using three reference sets

*R*^2^ threshold^a^	Reference set		
	ALL	EUR	MER
*Considering all variants tested in each reference set* ^b^			
thr0	0.935 (10.8)	0.938 (10.2)	0.918 (7.9)
thr4	0.965 (7.1)	0.962 (7.5)	0.963 (5.8)
*Considering only the variants tested in MER reference set* ^c^			
thr0	0.924 (7.9)	0.929 (7.9)	0.918 (7.9)
thr4	0.962 (6.3)	0.959 (6.7)	0.963 (5.8)

ALL = all 935 animals representing breeds from Europe, Africa, Asia, America and the Middle East; EUR = 726 animals representing breeds from Europe; and MER = 117 Merino animals

^a^thr0 and thr4 refer to Minimac3 *R*^2^ thresholds 0 and > 0.4, respectively

^b^Total number of imputed variants in each reference set for OAR1, 5, 10, 15, 20 and 25 in brackets

^c^All scenarios evaluated only on the same variants (30,660,937) in MER reference set

We found little difference in the imputation accuracy between using the ALL or EUR reference sets (the ALL set represented only 209 extra animals of non-European breeds). Although the largest number of variants was observed using the ALL reference set, after imposing the *R*^2^ thr4 filter, the largest number of variants was observed when using the EUR reference set (0.4 M more than when using the ALL reference set). The imputation accuracy for these two reference sets was similar when we evaluated accuracy for only the set of WGS variants that were imputed in the MER reference set.

**Fig. 4:**
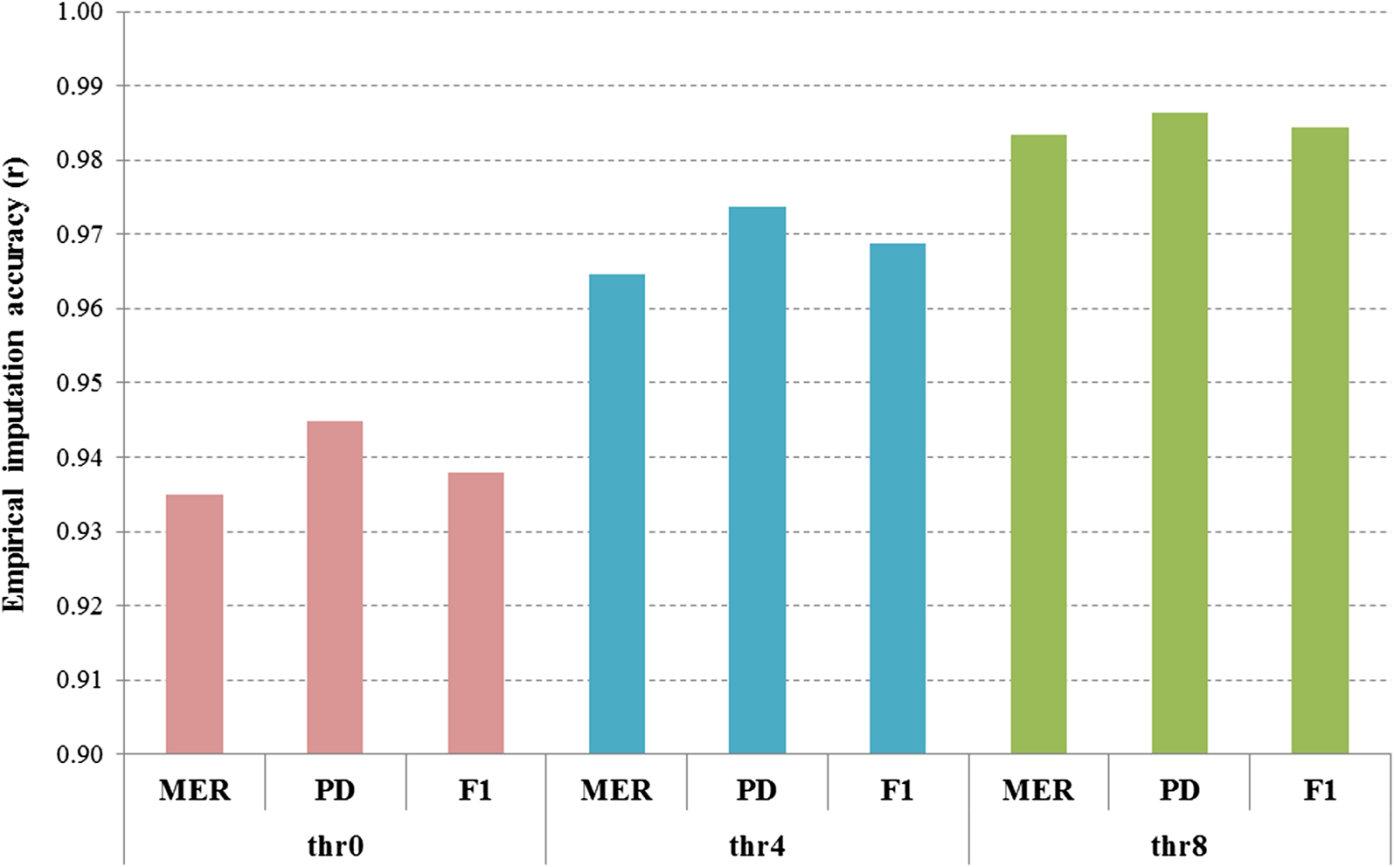
Empirical imputation accuracy of whole-genome sequence genotypes (Minimac3 most likely genotypes) in Merino (MER), Poll Dorset (PD), and F1 Merino X Border Leicester (F1), using a mixed breed reference population (ALL). Imputation accuracy is before and after imposing Minimac3 *R*^2^ thresholds to filter the imputed sequence data: thr0 = no threshold, thr4 =* R*^2^ > 0.4, and thr8 = *R*^2^ > 0.8. Imputation accuracy was measured as the correlation between imputed and observed sequence genotypes

Figure 4 compares imputation accuracies for the MER (Merino), PD (Poll Dorset) and F1 (Border Leicester x Merino) target populations (MM012) with the ALL reference set. Although the number of pure PD was small in the reference set (N = 29 minus 6 used for cross-fold validation) compared to MER (reference set N = 94), the accuracy in PD was always higher than in MER (Fig. 4). The accuracy for the F1 population (reference set N = 48) was lower than for PD, but was similar to that for MER. These differences in imputation accuracy between target sets remained regardless of the *R*^2^ threshold used. The average increase in imputation accuracy across target populations was 3% from thr0 (no filter) to thr4 (*R*^2^ > 0.4) compared to only 1.5% from thr4 to thr8 (*R*^2^ > 0.8) (Fig. 4). The average accuracy across target populations varied from 0.93 (for thr0) to 0.98 (for thr8). We also found no differences in imputation accuracies in the PD and F1 target sets when using either the EUR or ALL reference sets (results not shown).

**Fig. 5:**
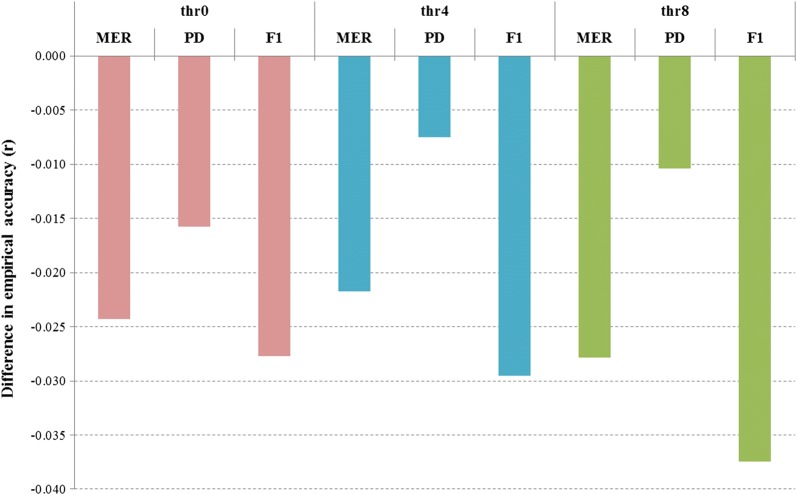
Difference in empirical imputation accuracy of whole-genome sequence genotypes (Minimac3 most likely genotypes) when imputation started from low-density SNP versus starting from high-density SNP chips. Accuracy was tested in three target sets: Merino (MER), Poll Dorset (PD) and Merino X Border Leicester crossbreds (F1), and the reference population was mixed European and non-European breeds (ALL). Comparisons are given before and after imposing Minimac3 *R*^2^ thresholds to filter the imputed sequence data: thr0 = no threshold, thr4 =* R*^2^ > 0.4, and thr8 = *R*^2^ > 0.8. Imputation accuracy was measured as the correlation between imputed and observed sequence genotypes

Next, we evaluated the accuracy of WGS imputation when starting from WGS pruned down to low-density SNP genotypes (OvineLD: ~ 12 k) compared to starting from WGS pruned down to high density (HD: ~ 500 k SNPs). Note that imputation from OvineLD to WGS genotypes was carried out stepwise: first to 50 k, then to HD and finally to WGS (see Methods). Figure 5 shows the difference in WGS imputation accuracy when starting from OvineLD genotypes compared with imputation starting directly from real HD genotypes. The results are shown for each target set at different *R*^2^ thresholds using the ALL reference set. For all target sets and *R*^2^ thresholds, accuracy was slightly lower when starting imputation from OvineLD genotypes compared to HD genotypes. The most marked difference was for the F1 crosses (approximately 3%) and the smallest difference was for PD (approximately 1%). However, after imposing filters on *R*^2^ thresholds, approximately 17% more variants were filtered out for imputation from OvineLD genotypes to WGS (regardless of the *R*^2^ threshold used) compared to imputation from real HD genotypes to WGS.

### Imputation accuracy across the genome

**Fig. 6:**
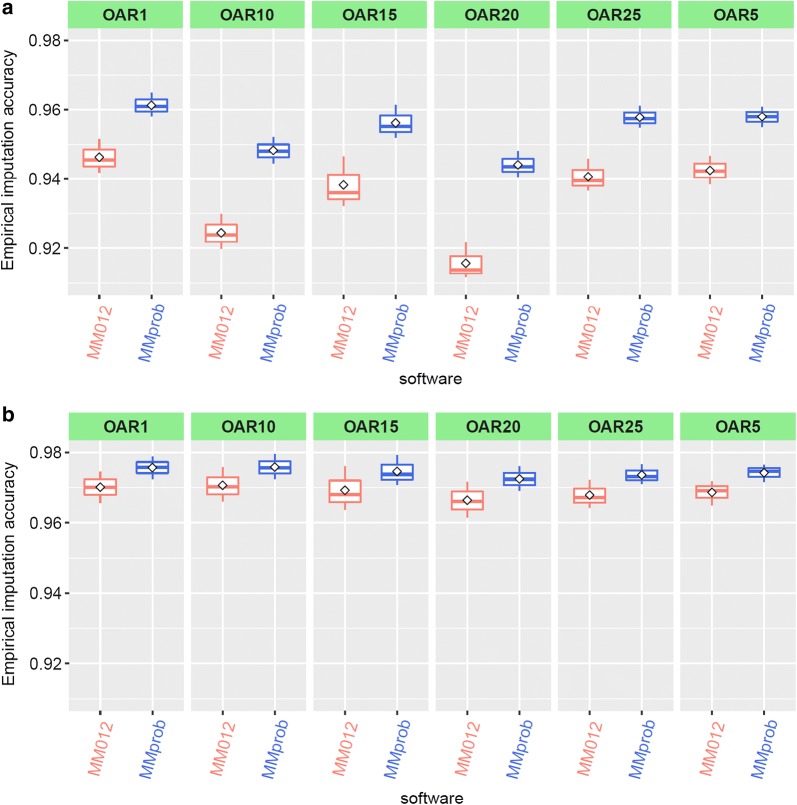
Mean empirical imputation accuracy of whole-genome sequence genotypes across target sets (MER, PD and F1) based on the Minimac3 (MM012) most likely genotypes and the Minimac3 allele dosage probability (MMprob) on six chromosomes (OAR1, 5, 10, 15, 20, 25). Imputation was implemented using a mixed European and non-European breed reference set (ALL). **a** provides imputation accuracy before imposing any Minimac3 *R*^2^ threshold to filter the imputed sequence data, while **b** provides imputation accuracy after applying a threshold of Minimac3 *R*^2^ > 0.4. Imputation accuracy was measured as the correlation between imputed and observed sequence genotypes

There were clear differences in average imputation accuracies across target sets between the six chromosomes (OAR) evaluated (Fig. 6a, for MMprob and MM012). These differences between chromosomes were considerably reduced after imposing the thr4 filter (Fig. 6b). The imputation accuracy was always highest for OAR1 and lowest for OAR10, with approximately 2.2% difference in accuracy between them without applying a *R*^2^ filter (thr0: Fig. 6a). After applying the thr4 filter, the accuracy improved for all chromosomes and the difference in accuracy between these two chromosomes (OAR1 and OAR10) decreased to only 0.05%. For all chromosomes evaluated, the accuracy of MMprob was consistently higher than that of MM012, and the difference between MM012 and MMprob decreased after imposing the thr4 filter (Fig. 6b).

**Fig. 7:**
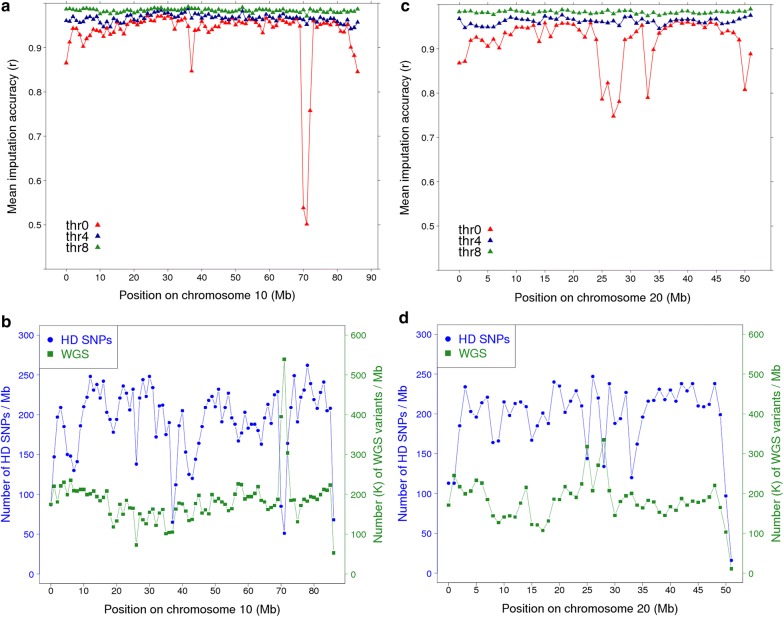
Mean empirical imputation accuracy of whole-genome sequence genotypes (Minimac3 most likely genotypes) based on non-overlapping 1-Mb windows along OAR10 (**a**) and 20 (**c**) before and after applying different thresholds based on the Minimac3 *R*^2^ statistic: thr0 = no threshold, thr4 = *R*^2^ > 0.4, and thr8 = *R*^2^ > 0.8. Imputation accuracy was measured as the correlation between imputed and observed sequence genotypes. **b** and **d** show the number of variants per Mb of HD (high-density) SNPs (blue) and imputed sequence variants (green) on OAR10 and 20. The region on OAR20 with low imputation accuracy (~ 25 – 28 Mb) coincides with the MHC region

To investigate these differences in accuracy between chromosomes further, we calculated the mean imputation accuracy for all variants within non-overlapping 1-Mb windows along each chromosome (MER target set with ALL reference set). We also calculated the density of both HD and WGS variants within the same 1-Mb windows to determine if the low imputation accuracy was more likely a result of poor HD SNP density and/or due to more highly polymorphic regions. A very poorly imputed 4 Mb region on OAR10 was identified between 70 and 74 Mb (Fig. 7a), that showed a low density of HD SNPs and a high density of WGS variants (Fig. 7b). We also discovered a region with very poor imputation accuracy, which coincided with the MHC region on OAR20 (at approximately 25–28 Mb, Fig. 7c). This same region had a low density of HD SNPs and a high density of WGS variants, which was an atypical pattern compared to the rest of the chromosome (Fig. 7d). Notably, OAR10 and 20 had the lowest overall imputation accuracies compared to OAR1, 5, 15 and 25 (Fig. 6a).

Several other very poorly imputed regions were also observed, on OAR4, 7, 14 and 21 (see Additional file 3: Figure S2) in the MER target set with the ALL reference set. The corresponding HD SNP and WGS variant densities across all autosomes are also provided in Figure S3 (see Additional file 4: Figure S3). As expected, the chromosome ends always tended to be less accurately imputed due to a lack of HD flanking haplotype information. After imposing the thr4 filter, the imputation accuracy increased to above 0.9 for all 1-Mb windows, except for one very poorly imputed region on OAR21 (see Additional file 3: Figure S2).

**Fig. 8:**
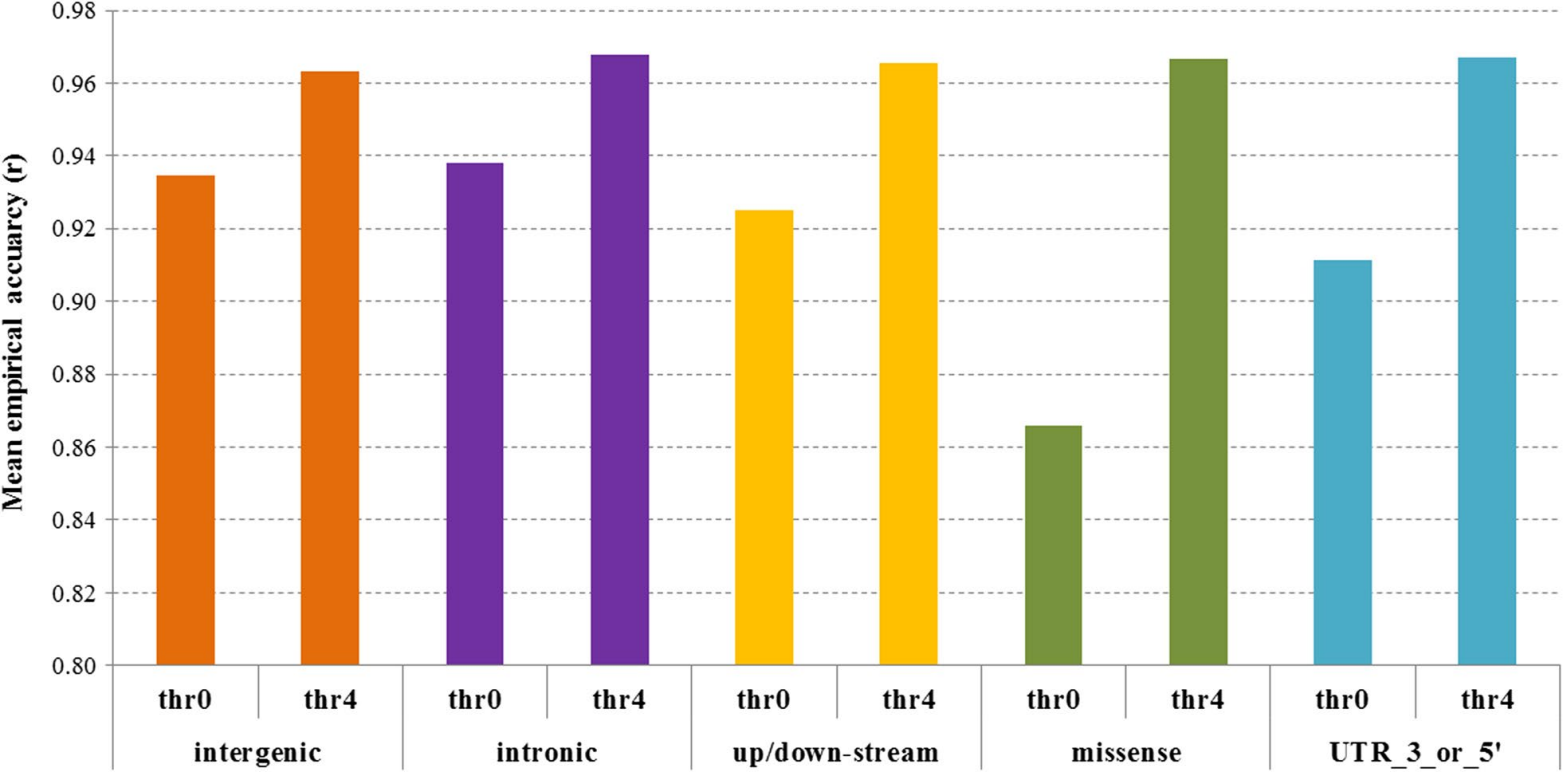
Empirical imputation accuracy of whole-genome sequence genotypes (Minimac3 most likely genotypes) variants in five genome annotation classes: intergenic, intronic, 5 kb up- or down-stream of genes, missense, and 3’ and 5’ UTR. Imputation was carried out in a Merino (MER) target set using a mixed European and non-European breed reference set (ALL). Imputation accuracy was measured as the correlation between imputed and observed sequence genotypes, before (thr0) and after filtering variants based on Minimac3 *R*^2^ > 0.4 (thr4)

We also compared the mean empirical imputation accuracy for WGS variants within each of five key annotation groupings: intergenic, intronic, 5 kb up- or down-stream of genes, missense, and UTR 3’ and 5’ ends (for the MER target set with the ALL reference set: Fig. 8). Most variants were intergenic (63%), followed by intronic (30%), while variants in regions 5 kb up- or down-stream of genes accounted for 6.4% of the total, missense for 0.3%, and those in 3’ or 5’ UTR for 0.3%. The mean empirical imputation accuracies for these annotation classes without imposing an *R*^2^ filter (thr0) ranged from 0.866 (missense) to 0.938 (intronic). After retaining the variants with *R*^2^ > 0.4 (thr4), the difference in empirical imputation accuracies between the annotation classes was much reduced (Fig. 8). However, the proportion of variants filtered out (*R*^2^ ≤ 0.4) was much higher in the missense class (65%) than in the intergenic class (35%).

**Fig. 9:**
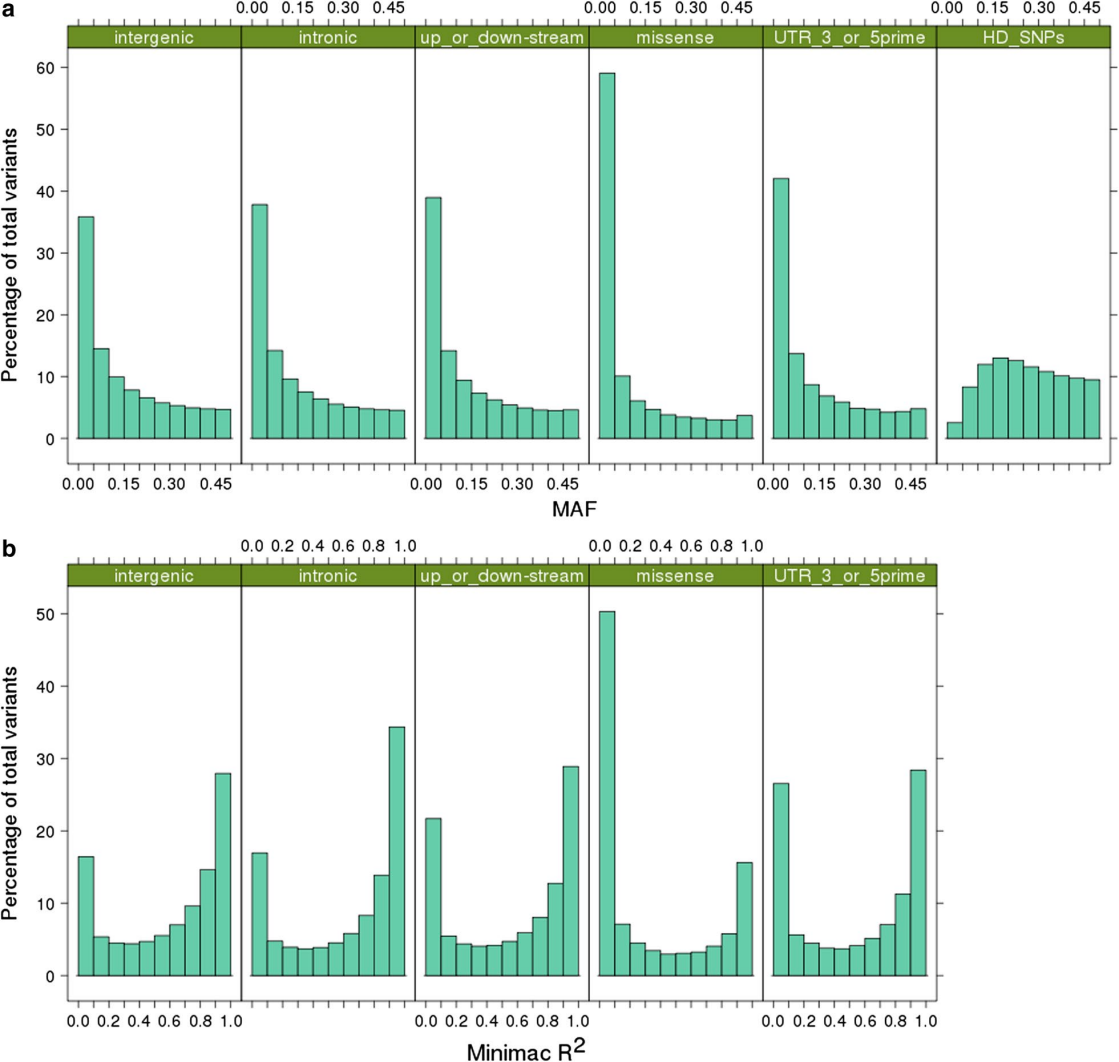
Distributions of minor allele frequency (MAF) (**a**) and Minimac3 *R*^*2*^ (**b**) of variants in five annotation categories: intergenic, intronic, 5 kb up- or down-stream of genes, missense, and 3’ and 5’ UTR. The distribution of MAF is also shown for SNPs on a standard HD (high-density) SNP chip. Imputation was carried out in the Merino (MER) target set using a mixed European and non-European breed reference set (ALL)

To investigate the differences in imputation accuracy between annotation classes further, we compared the MAF and *R*^2^ distributions for each annotation class (Fig. 9). All five annotation classes show a MAF profile that was highly skewed towards low MAF compared to the relatively uniform MAF distribution of HD SNP genotypes (Fig. 9a). The proportion of low MAF variants (< 0.05) was highest for the missense (approximately 60%) and UTR variants (approximately 42%), and this was clearly reflected in the *R*^2^ distribution, for which the proportion of variants with *R*^2^ ≤ 0.4 was also highest for the missense and UTR variants (Fig. 9b). The *R*^2^ had a U-shaped distribution for all annotation classes, with an overrepresentation of high and low *R*^2^ (Fig. 9b). However, the difference in the proportion of variants with very low *R*^2^ (< 0.1) and the proportion of variants with very low MAF (< 0.05) was the highest for the missense variants and lowest for the intergenic and intronic variants.

### Impact of imputation accuracy (Minimac3 *R*^2^) on genomic prediction

Generally, the empirical accuracy of imputed variants is unknown, but we have demonstrated that the Minimac3 *R*^2^ is a reasonable proxy and can be used to filter out poorly imputed variants. Therefore, we imputed a large multi-breed sheep population to WGS and then evaluated the impact of filtering WGS variants using the Minimac3 *R*^2^ on the proportion of genetic variance explained by SNPs (“genomic heritability”) and on the accuracy of genomic prediction. Accuracies of GEBV were assessed using 50,000 sequence variants (MAF > 0.01) that were selected using three *R*^2^ thresholds: *R*^2^ ≤ 0.4 (thr0-4), *R*^2^ > 0.4 (thr4), and *R*^2^ > 0.8 (thr8).

**Fig. 10:**
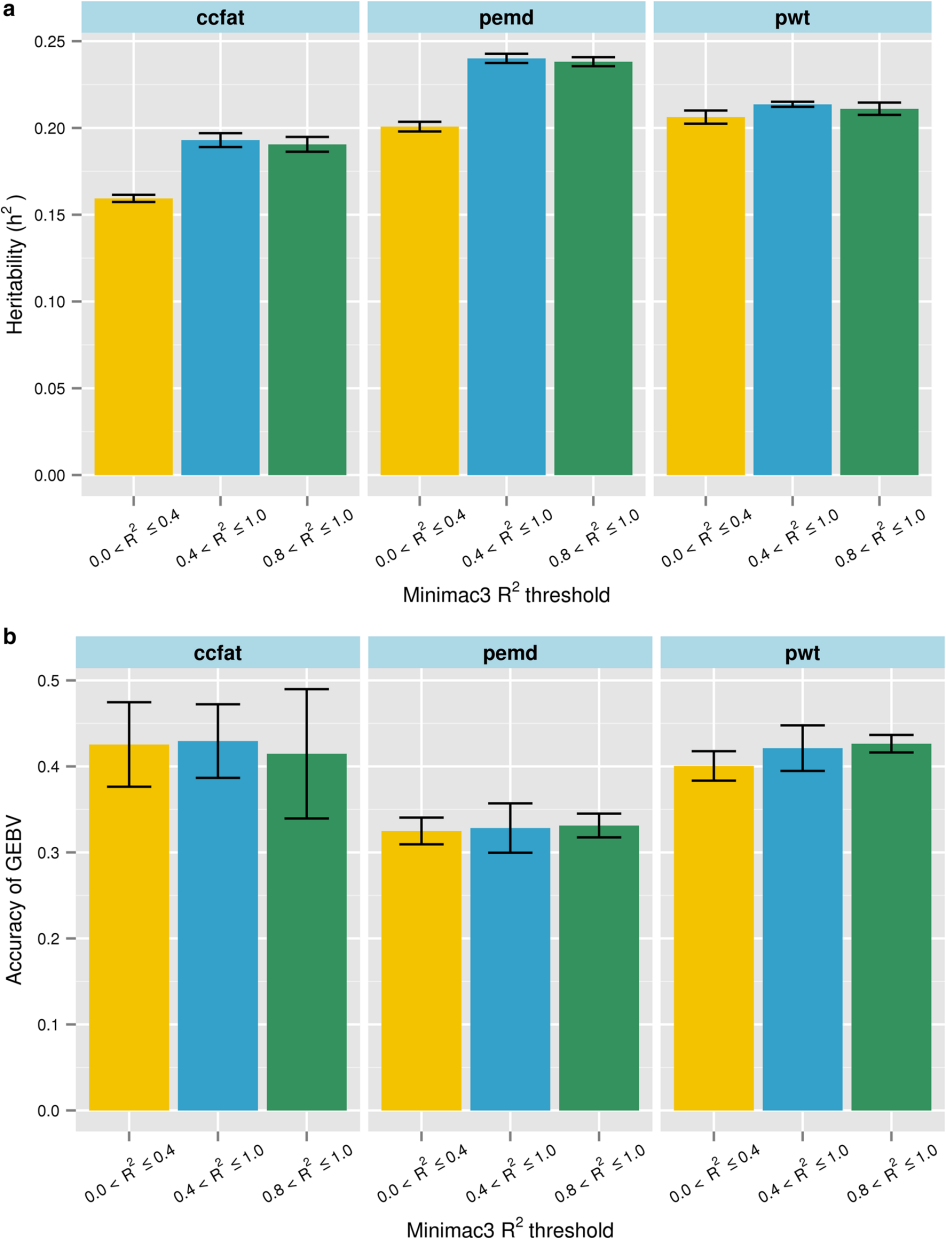
Mean genomic heritability estimates (**a**) and mean accuracies of genomic estimated breeding values (GEBV) (**b**) in a Merino validation population. Heritability estimates were based on a random selection of 50,000 imputed variants from three classes based on Minimac3 *R*^2^: 0.0 < *R*^2^ ≤ 0.4, 0.4 < *R*^2^ ≤ 1.0, and 0.8 < *R*^2^ ≤ 1.0

Comparing the genomic heritability results for the poorly imputed (thr0-4) with more confidently imputed (thr8) variant sets, genomic heritability clearly tended to be lower for the poorly imputed variants (Fig. 10a). There was no difference in genomic heritability observed between using thr4 and thr8, which might be expected given the “U” shaped distribution of *R*^2^ (Fig. 9b) because there was a very low proportion of WGS variants with 0.4 < *R*^2^ < 0.8 and, thus, most variants in thr4 had an *R*^2^ > 0.8. The genomic prediction accuracies, however, presented a less clear picture, with only pwt showing increased accuracy after imposing a higher filter threshold based on *R*^2^, possibly because we used a mixed breed reference population with a density of only 50 k variants and with most variants having a low MAF. As a result, the prediction accuracy was relatively low regardless of the *R*^2^ threshold applied because SNP effects were imprecisely estimated due to low LD between SNPs and causal variants.

## Discussion

This is the first study to evaluate the empirical accuracy of imputed whole-genome sequence variants in sheep, using up to 935 sequenced sheep as a mixed breed reference population. We present results for two commonly used imputation programs, for single- and multi-breed reference populations, and for one crossbred and two purebred target populations. The results provide confidence that imputation to WGS in sheep can achieve high accuracy using this mixed breed reference population, even when imputing from a low-density 12 k SNP array. These reference sequences are publicly available and, thus, our findings are widely applicable and provide a practical and cost-effective approach for imputing large mixed or single-breed sheep populations to WGS. We also demonstrated that it was practical to use Eagle and Minimac3 to phase and impute more than 47,000 mixed breed sheep to WGS and to use Minimac3 *R*^2^ to filter variants for genomic prediction.

### Imputation software

In our study, the FImpute and Minimac3 softwares performed equally well for imputing the most likely genotypes, which is in line with results for cattle [16]. Unlike Pausch et al. [16], we did, however, not provide FImpute pedigree information because sheep pedigrees are generally shallow and may also contain more errors than dairy cattle pedigrees, which could negatively impact imputation [26]. In addition, Minimac3 requires pre-phasing of haplotypes and is more computationally demanding than FImpute [16]. However, Minimac3 did not fail on any of the ovine 26 autosomes for this study, while FImpute failed to impute OAR10 without removal of a 4 Mb segment between 70 and 74 Mb. A similar issue was reported by Pausch et al. [16] when imputing cattle to WGS with FImpute. Typically, the regions that will be difficult to impute are not known a priori and, therefore, it can take considerable computational time to identify the genomic region that causes the issue when the program aborts. To assist with this, we provide a genome-wide map of regional imputation accuracy by 1-Mb segments (see Additional file 3: Figure S2), which can be used to troubleshoot if imputation software aborts when imputing an entire chromosome.

In contrast to FImpute, Minimac3 imputed the entire OAR10 chromosome without aborting but with an extremely low accuracy in the region where FImpute failed (Fig. 6). Thus, it is imperative to have a means of filtering out very poorly imputed variants because they may affect downstream analyses such as association studies or genomic prediction. Minimac3 provides an *R*^2^ quality statistic, which as we have demonstrated, can be used as a practical filter to remove poorly imputed variants (e.g. Minimac3 *R*^2^ > 0.4 corresponds to a mean empirical accuracy of 0.86, Fig. 3), although it is not a perfect predictor of empirical imputation accuracy. The most suitable *R*^2^ threshold to use depends on the type of downstream analyses. For example, there may not be an optimal threshold when searching for a gene with a major effect, but a modest threshold may be prudent when using imputed sequence for genomic prediction. In a recent genomic prediction study in sheep, we removed WGS variants with an *R*^2^ threshold of ≤ 0.4 and demonstrated that imputed sequence variants increased the accuracy of genomic prediction compared to using only the standard 50 k ovine SNP set [3, 8].

### Reference and target populations

Our results support the use of combining smaller purebred sequenced populations into a larger mixed breed reference set to impute both purebred and crossbred individuals, in agreement with findings for imputing purebred dairy cattle [16, 27]. Furthermore, this is expected in sheep because previous research demonstrated that there is widespread sharing of relatively large haplotypes across a large range of European breeds [28]. This could also explain why there was no further increase in accuracy using the ALL reference (N = 935) compared to the EUR set (N = 726). The ALL set included breeds from Asia and Africa that are known to diverge from European breeds [28]. Combining smaller sets of sequenced animals from different breeds into one large reference population provides a cost-effective approach to impute to WGS for sheep, particularly in countries such as Australia where many breeds and composites are used in the industry.

The imputation accuracies achieved in this study were similar to or higher than those reported for imputed WGS in dairy cattle [16, 27, 29]. This may be due to closer relationships (i.e. more haplotype sharing) between some animals in the target and reference sets compared to the cattle studies where 1000 bulls were more distant relatives [2]. In addition to the purebreds, our EUR set included 177 Australian sheep that were crossbreds (“composites”): more than 90% of these animals had some recent Merino ancestry, 63% had some recent Border Leicester ancestry and a lower proportion included some recent Poll Dorset (37%) and White Suffolk (30%) ancestry. The White Suffolk breed in Australia was developed initially by crossing Suffolk to mainly Poll Dorset to remove the dark pigmented body regions. The target populations used in this study represent wool (Merino, MER), terminal (Poll Dorset, PD) and maternal breeds (Border Leicester F1 cross) that have quite different effective population (Ne) sizes: estimated as 833 for MER, 318 for PD and 242 for BL, respectively [28]. This likely explains why the imputation accuracy was always higher in the PD and F1 animals compared to the MER population although there were fewer PD and Border Leicester animals in the reference population (both pure and crossbreds). This phenomenon was also observed in an imputation study from low- to medium-density SNPs in sheep [22].

### Regional imputation accuracy across the genome

In agreement with recent findings in cattle [16], we provided evidence that the mean imputation accuracy differed across chromosomes largely as a result of one or more substantial genomic regions (≥ 1 Mb) being poorly imputed (e.g. a 4 Mb region on OAR10 at around 70 Mb). This indicates a regional mapping issue because the accuracy was averaged across 1-Mb windows, which would not be influenced by an occasional poorly imputed variant. In addition, the HD SNP density was generally low in these regions and in some regions, there was also a higher than usual density of sequence variants (e.g. OAR10 around 70 Mb). This could arise for example, from alignment errors due to large segmental duplications. In dairy cattle, Pausch et al. [16] also found a sharp decline in imputation accuracy in several regions of the bovine genome that were previously identified as containing large tandem segmental duplications.

Alternatively, the increased density of sequence variants could also reflect a truly more polymorphic region resulting in the region being more challenging to impute than less highly polymorphic regions. For example, the MHC region on OAR20 (around 25–28 Mb, [30]) showed poor imputation accuracy and a higher density of polymorphic sequence variants. The MHC region in sheep and cattle is known to harbour some duplicated regions [30] and also in humans is generally found be highly polymorphic due to strong selection by evolutionary pressures such as many different pathogens [31]. The ovine MHC region shows a very strong homology with the bovine MHC [30], thus it is not surprising that this region is also poorly imputed in cattle [16]. Liu et al. [32] emphasized that low heterozygosity, high sequence similarity to other genomic regions, high GC content, segmental duplication and distance between genotyped markers are the major contributing factors to consistently poor imputation.

The Minimac3 *R*^2^ statistic and MAF of annotated variants suggest purging selection on missense variants because these variants have the highest proportion of variants in the lowest MAF band. Furthermore, the higher relative proportion of low *R*^2^ (< 0.1) to low MAF (< 0.05) variants in missense compared to intergenic and intronic categories suggests poorer imputation accuracy overall in missense variants. This could indicate that the missense variants are often younger than variants in the more neutral categories (due to purging selection) and younger mutations can be more difficult to impute accurately because the surrounding haplotypes are present with and without the new mutation [33].

### Genomic prediction

The estimates of genomic heritability (Fig. 10) suggest that using the Minimac3 *R*^2^ statistic to pre-filter WGS variants before selecting subsets for downstream studies such as genomic prediction or association studies is a sensible strategy. The Minimac3 *R*^2^ calculation is based on the premise that poorly imputed allele counts are shrunken towards their expectations based on the estimated allele frequency. Imputation for genomic prediction was carried out in a mixed breed target set, rather than single-breed target set, and thus it is possible that the *R*^2^ statistic was a less precise predictor of imputation quality for the mixed breed set than for the single breed set. This, in turn, may have influenced our genomic prediction results, for which there was no clear disadvantage for the low *R*^*2*^ variants. Within the scope of this study, it was not possible to compare our genomic prediction results with results from a ‘gold standard’ set of real 50 k genotypes for two reasons: first, many animals in this population were only genotyped on LD SNP arrays and, second, the MAF distribution of SNPs on the 50 k chip is very different than the distribution of MAF for the random sets of SNPs selected here.

Druet et al. [34] demonstrated that the use of WGS variants for genomic prediction (compared to dense SNP panels) provides the largest increase in accuracy when the causal sequence variants are rare and thus in low LD with the SNPs genotyped on panels. For example, in our data, the missense variants had a much higher proportion of rare variants than HD array SNPs and are also more likely to affect phenotypes because they change the protein coding sequence. However, these low MAF variants are also more likely to be poorly imputed and they comprise a large proportion of sequence variants (~ 65% with *R*^2^ < 0.4; Fig. 9). Thus, it is useful to have a means of filtering out the least accurately imputed variants, such as by applying the Minimac3 *R*^2^ statistic. However, it is also important to enlarge the reference population such that there is more information to impute the less common variants more accurately.

## Conclusions

We achieved a mean accuracy of imputation to whole-genome sequence of up to 0.97 across target breeds using the Minimac3 software, with pre-phasing using the Eagle software and filtering variants based on the Minimac3 *R*^2^ being higher than 0.4. Imputation accuracy improved by combining smaller breed sets into a multi-breed reference. The empirical accuracy varied based on MAF, target breed, reference breed composition, and chromosome region.

## Additional files

**Additional file 1.** Breed names and number of sequenced animals from each breed in ALL and EUR reference sets.

**Additional file 2.** Allele distribution for ccfat for each Minimac3 *R*^2^ filter (0.0 < *R*^2^ ≤ 0.4, 0.4 < *R*^2^ ≤ 1.0, and 0.8 < *R*^2^ ≤ 1.0) with five random sets of 50,000 genome-wide variants.

**Additional file 3.** The empirical imputation accuracy across ovine autosomes using ALL reference and MER target set.

**Additional file 4.** HD SNP and WGS variant density across ovine autosomes using ALL reference and MER target set.
