# Supplementary figures and images for: Accuracy of imputation to whole-genome sequence in sheep
Source: Genet Sel Evol. 2019 Jan 17;51:1. doi: 10.1186/s12711-018-0443-5 (PMC6337865; doi:10.1186/s12711-018-0443-5)

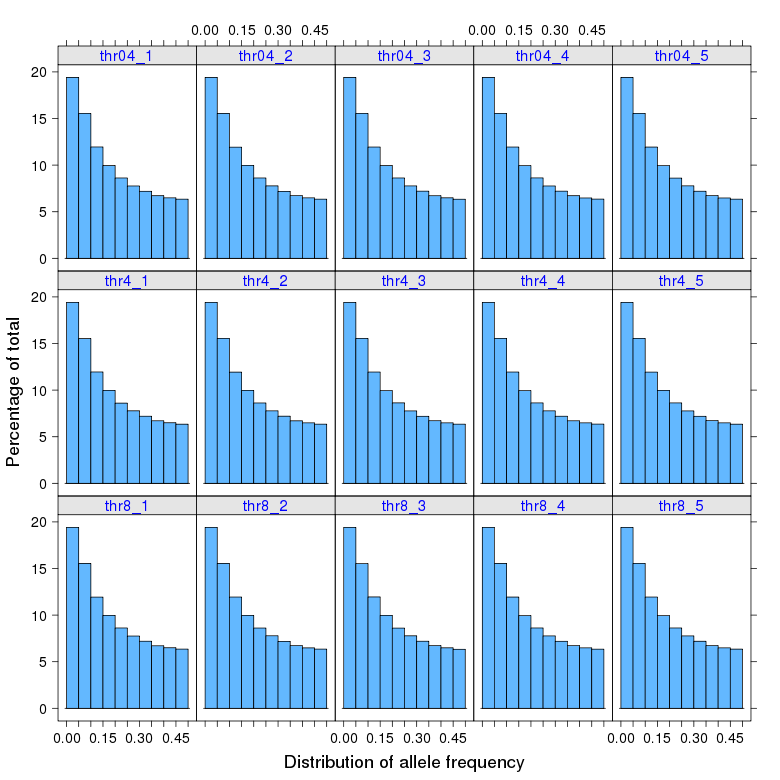

Supplement: Supplementary file 2 — Additional file 2. Allele distribution for ccfat for each Minimac3 R2 filter (0.0 < R2 ≤ 0.4, 0.4 < R2 ≤ 1.0, and 0.8 < R2 ≤ 1.0) with five random sets of 50,000 genome-wide variants. [file 12711_2018_443_MOESM2_ESM.png]

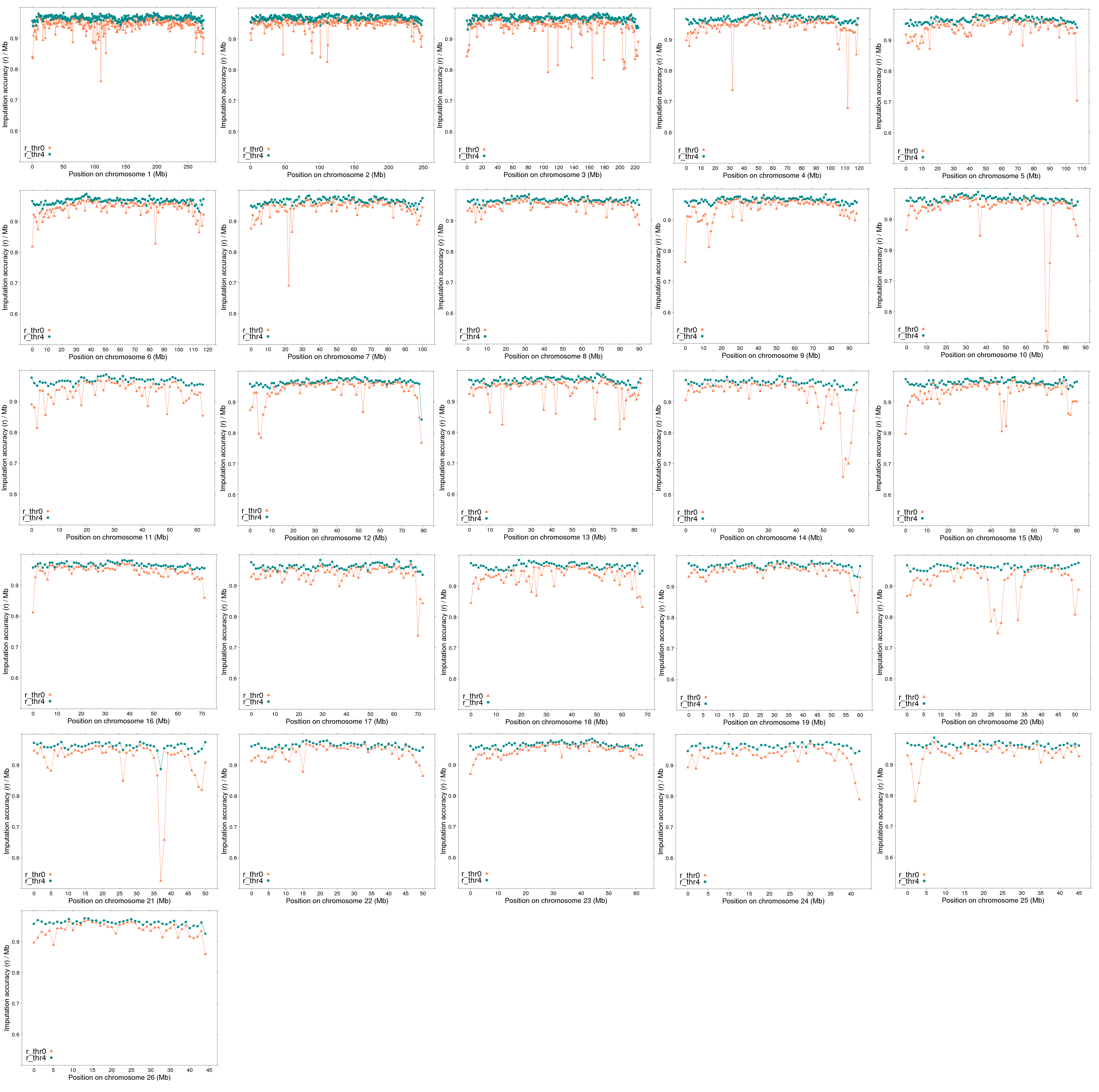

Supplement: Supplementary file 3 — Additional file 3. The empirical imputation accuracy across ovine autosomes using ALL reference and MER target set. [file 12711_2018_443_MOESM3_ESM.png]

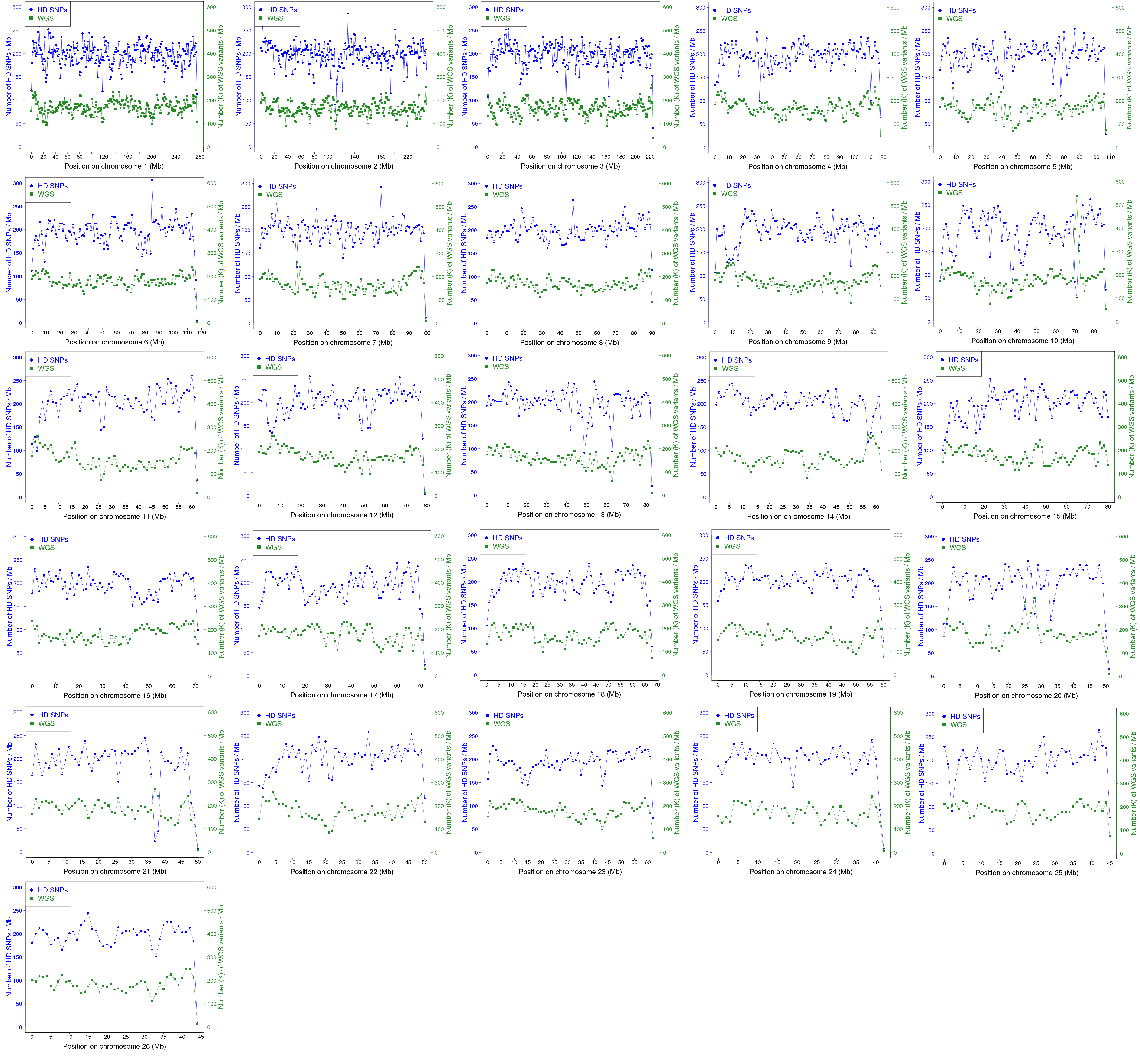

Supplement: Supplementary file 4 — Additional file 4. HD SNP and WGS variant density across ovine autosomes using ALL reference and MER target set. [file 12711_2018_443_MOESM4_ESM.png]
